# Total Cystectomy in the Management of Primary Retroperitoneal Echinococcal Cysts: Current Evidence and Future Directions

**DOI:** 10.7759/cureus.57218

**Published:** 2024-03-29

**Authors:** Mazin M Almomani, Faisal F Al-shaikhly, Rahaf T Oduibat, Abedallah J Al-Adwan, Lina M Al-Tarawneh

**Affiliations:** 1 Medicine and Surgery, University of Jordan, Amman, JOR; 2 Medicine and Surgery, Jordan University Hospital, Amman, JOR; 3 General Practice, Jordanian Royal Medical Services, Amman, JOR; 4 General Practice, Jordan University Hospital, Amman, JOR

**Keywords:** future directions, adjuvant therapies, minimally invasive approaches, complications, recurrence rates, systematic review, surgical options, management, total cystectomy, retroperitoneal echinococcal cysts

## Abstract

Echinococcal cysts (ECs) are a significant public health concern globally, particularly in endemic regions. Among these, primary retroperitoneal echinococcal cysts (PRECs) present unique challenges due to their location and complex presentations. Total cystectomy, involving complete removal of the EC and surrounding tissue, is a surgical option for managing PRECs. However, evidence regarding its efficacy and safety is limited. We conducted a systematic review following Preferred Reporting Items for Systematic Reviews and Meta-Analyses (PRISMA) guidelines to evaluate the role of total cystectomy in managing PRECs. A comprehensive search of databases yielded four relevant studies. These studies reported favorable outcomes following total cystectomy, including low recurrence rates and minimal postoperative complications. However, challenges such as technical complexity and proximity to vital structures were noted. Future research should focus on evaluating minimally invasive approaches, exploring adjuvant therapies, identifying predictors of recurrence, and assessing cost-effectiveness. This systematic review underscores the need for further investigation to optimize the management of PRECs and improve patient outcomes.

## Introduction and background

Echinococcal cysts (ECs), caused by parasite infections of the genus Echinococcus, pose a significant health threat in various regions globally. These cysts can develop in different organs, with the liver and lungs being the most common sites [1]. However, retroperitoneal echinococcal cysts (RECs), though rare, present unique challenges in management due to their anatomical location and potential for complex presentations. The prevalence of ECs varies considerably across geographical regions, with the highest burdens observed in areas with sheep-dog cycles, such as South America, Central Asia, and parts of the Mediterranean and East Africa [2]. Estimates suggest that millions of individuals worldwide harbor these cysts, potentially leading to significant morbidity and mortality if left untreated [2].

Among the various surgical options, total cystectomy, involving complete removal of the EC and surrounding host tissue, has been explored for the management of primary retroperitoneal echinococcal cysts (PRECs). This approach aims to achieve definitive parasite eradication and minimize the risk of recurrence [3]. While other surgical techniques, such as partial resection or unroofing, exist, total cystectomy presents potential advantages in terms of potentially lower recurrence rates and improved long-term outcomes [3]. The rationale for investigating the role of total cystectomy in the management of PRECs stems from several factors. Firstly, the anatomical complexity of the retroperitoneal space can impede complete parasite removal through less invasive techniques, potentially increasing the risk of recurrence. Secondly, the potential for involvement of vital structures in this region necessitates a thorough approach to minimize the risk of postoperative complications. Additionally, concerns regarding albendazole resistance in certain Echinococcus species highlight the need for definitive surgical solutions in specific cases.

Despite the potential benefits of total cystectomy, limited high-quality evidence exists to definitively establish its effectiveness and safety in this specific context. Previous studies have reported varying outcomes and employed diverse surgical techniques, making it difficult to draw definitive conclusions. Therefore, a systematic review of the available literature is necessary to comprehensively evaluate the available evidence and provide informed guidance on the role of total cystectomy in this patient population.

## Review

Materials and methods

Search Strategy

This systematic review follows the Preferred Reporting Items for Systematic Reviews and Meta-Analyses (PRISMA) guidelines. A meticulous investigation of pertinent literature spanned renowned databases recognized for their comprehensive medical and scientific reports, namely PubMed, Embase, Web of Science, and Scopus. These databases were chosen for their extensive collection of peer-reviewed articles, ensuring a robust foundation for our systematic review of the management of PRECs through total cystectomy. Our search employed a carefully curated set of keywords and phrases aligned with the study's objectives, encompassing terms such as "Total Cystectomy," "Primary Retroperitoneal Echinococcal Cysts," and related expressions. Boolean operators "AND" and "OR" were strategically deployed to formulate a comprehensive search algorithm. For instance, the string "Total Cystectomy AND Primary Retroperitoneal Echinococcal Cysts" focused on studies explicitly addressing the management of these cysts through total cystectomy. The use of "OR" facilitated the inclusion of broader terms associated with the topic, ensuring an exhaustive exploration of the literature.

To maintain a contemporary and relevant scope, our search was confined to studies published from the inception of each database to January 2024. This timeframe allowed for the inclusion of historical and current research, offering a comprehensive overview of the role of total cystectomy in managing PRECs. Filters were applied to include studies in the English language and those involving human subjects, aligning with the specific objectives of our review. Additionally, manual searches of the reference lists of included studies and relevant reviews were conducted to ensure a thorough exploration of the literature. This rigorous and expansive search strategy aimed to capture the full spectrum of evidence concerning the role of total cystectomy in the management of PRECs, providing a robust foundation for our systematic review.

Eligibility Criteria

In determining eligibility criteria for our systematic review on the management of PRECs through total cystectomy, precision and relevance were paramount. The types of studies included comprised peer-reviewed research articles, observational studies, and clinical trials, reflecting a commitment to high-quality, evidence-based knowledge. Our review encompassed studies investigating the efficacy of total cystectomy, with outcomes such as recurrence rates, complications, and overall patient outcomes considered. To ensure comprehensiveness, studies published in the English language were included, acknowledging English as the predominant language of scientific communication. The inclusion timeframe extended from the inception of the respective databases to the present date, allowing for the synthesis of contemporary research on the management of PRECs through total cystectomy.

Conversely, exclusion criteria were carefully tailored to maintain the focus and methodological rigor of the review. Studies not directly addressing the role of total cystectomy in the management of PRECs, as well as those lacking relevant outcome measures, were omitted. Non-English language publications, unpublished works, and gray literature such as conference abstracts were also excluded. Furthermore, studies presenting insufficient data on the role of total cystectomy in the management of PRECs were excluded, aligning with the review's commitment to human-centered, data-rich research.

Data Extraction

The data extraction process for our systematic review of the management of PRECs through total cystectomy was meticulously structured to ensure the integrity of our research findings. This critical process unfolded in two stages, emphasizing thoroughness and accuracy. In the initial stage, a preliminary screening was conducted to filter articles based on relevance indicated by titles and abstracts. Two independent reviewers performed this assessment, classifying each article as relevant, not relevant, or probably relevant based on a conscientious appraisal of the study's abstract and its pertinence to the review's focus. Progressing to the second stage, full-text articles deemed relevant or probably relevant underwent a detailed examination. Two independent reviewers utilized a standardized data extraction template within Microsoft Excel to capture and organize critical information. Both reviewers independently applied pre-established inclusion and exclusion criteria to each study, with any discrepancies resolved through the adjudication of a third independent reviewer. This meticulous data extraction process ensured that all relevant data points were captured, contributing to the robustness and credibility of our systematic review's conclusions.

Results

Study Selection Process

**Figure 1:**
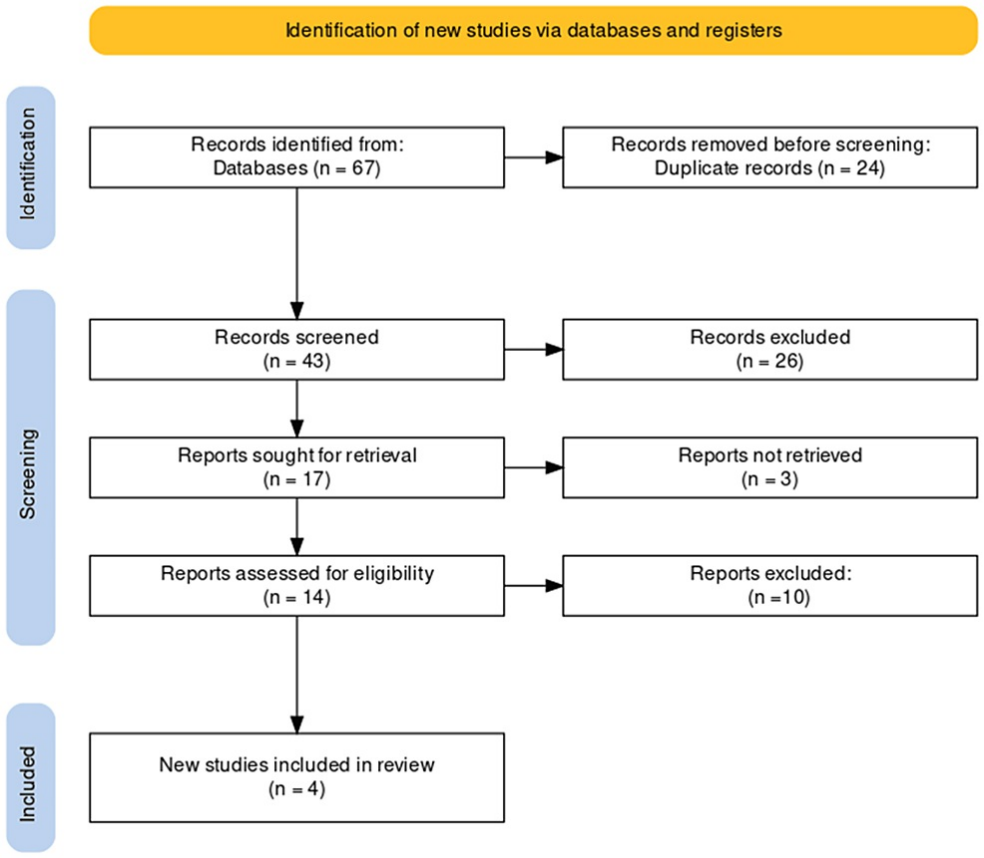
Preferred Reporting Items for Systematic Reviews and Meta-Analyses (PRISMA) flow diagram of the selection of studies for inclusion in the systematic review.

The study selection process for our systematic review on the management of PRECs through total cystectomy adhered to the PRISMA guidelines, ensuring a transparent and systematic approach. A comprehensive search across databases initially yielded 67 studies, from which 24 duplicates were removed, resulting in a refined pool of 43 unique studies. Subsequent examination of titles and abstracts led to the exclusion of 26 records that did not meet predefined relevance criteria. A rigorous evaluation of the remaining 17 articles, involving retrieval and scrutiny of full texts, led to the exclusion of 13 reports that did not align with stringent inclusion criteria. The culmination of this meticulous selection process identified four studies deemed suitable for inclusion in our review, providing a focused and rich source of evidence for the analysis of the role of total cystectomy in managing PRECs. The PRISMA flowchart, detailing the study selection process, is presented in the following figure (Figure 1). The key details of each selected study are tabulated in the following table (Table 1).

**Table 1:** A summary of the studies included in this systematic review.

Author	Year	Country	Study Type	Number and gender of participants	Location	Size (cm)	Preoperative intervention	Surgical intervention	Mean Operative time (mins)	Post-operative complications	Post-operative hospital stay (days)	Recurrence
Mehri et al. [4]	2022	Iran	Case Report	n = 1; 22-year-old male	Retroperitoneal cyst between diaphragm and bladder	20 x 14	Albendazole	Total cystectomy	N/A	None reported	3	None after 6 months followup
Ozturk et al. [5]	2014	Turkey	Case Report	n = 1; 43-year-old female	Retroperitoneal cyst situated among the left liver lobe, diaphragm, spleen, tail of the pancreas, and transverse colon, with invasion into the splenic hilum	17 x 11	None	Total cystectomy and spleenectomy	150	None reported	7	None after 3 months followup
Gavriilidis et al. [6]	2012	Greece	Case Report	n = 1; 74-year-old male	Retroperitoneal multiseptated cyst containing daughter cysts without calcifications	N/A	Albendazole 400 mg twice daily for 1 month	Total cystectomy and right hemicolectomy	N/A	None reported	N/A	None
Tepetes et al. [7]	2007	Greece	Case Report	n = 1; 28-year-old female	Retroperitonal cyst firmly attached to sacrum and in contact with the left ovary and left ureter	9 x 4	None	Total csytecomy with a portion of mesosigmoid	N/A	None reported	6	None after 3 months followup

Quality Assessment

The quality assessment of the selected studies for this systematic review was conducted using the Joanna Briggs Institute (JBI) critical appraisal checklist for case reports. The assessment results indicated the quality of each study, providing insights into the reliability and validity of the evidence presented (Table 2).

**Table 2:** Quality assessment of the included studies using Joanna Briggs Institute (JBI) critical appraisal checklist for case reports.

JBI critical appraisal checklist for case reports	Mehri et al. [4]	Ozturk et al. [5]	Gavriilidis et al. [6]	Tepetes et al. [7]
Were patient’s demographic characteristics clearly described?	Yes	Yes	Yes	Yes
Was the patient’s history clearly described and presented as a timeline?	Yes	Yes	Yes	Yes
Was the current clinical condition of the patient on presentation clearly described?	Yes	Yes	Yes	Yes
Were diagnostic tests or assessment methods and the results clearly described?	Yes	Yes	Unclear	Yes
Was the intervention(s) or treatment procedure(s) clearly described?	Yes	Yes	Yes	Yes
Was the post-intervention clinical condition clearly described?	Yes	Yes	Yes	Yes
Were adverse events (harms) or unanticipated events identified and described?	Yes	Yes	Yes	Yes
Does the case report provide takeaway lessons?	Yes	Yes	Yes	Yes

Discussion

Echinococcosis, caused by the larval stage of the Echinococcus tapeworm, remains a significant public health concern globally, particularly in endemic regions [8,9]. Among the various manifestations of echinococcosis, PRECs pose unique diagnostic and management challenges due to their location and presentation. ECs can be classified based on their location as either primary or secondary. Primary cysts develop from the larval stage of the Echinococcus tapeworm in the intermediate host, typically humans, following ingestion of contaminated food or water. Secondary cysts, on the other hand, result from the rupture of a primary cyst, leading to the dissemination of daughter cysts to other organs, including the retroperitoneum [10,11]. PRECs originate from the larval stage of Echinococcus granulosus or Echinococcus multilocularis primarily within the retroperitoneal space, often adjacent to the liver or spleen [12]. These cysts can grow to significant sizes and may remain asymptomatic for prolonged periods, making their diagnosis and management challenging. In contrast, secondary retroperitoneal echinococcal cysts (SRECs) are far more common and arise from the rupture or seeding of cysts originating in the liver, lungs, or other visceral organs.

The clinical presentation of PRECs can vary widely depending on the size, location, and involvement of adjacent structures. Patients may present with nonspecific symptoms (such as abdominal discomfort, dull pain, or a palpable mass in the abdomen) owing to the slow growth and space-occupying nature of these cysts. As the cyst grows, it may compress surrounding organs, leading to obstructive symptoms such as urinary retention or bowel obstruction. Constitutional symptoms such as fever, weight loss, and fatigue may be present in advanced cases. In rare cases, rupture of the cyst may occur, resulting in potentially life-threatening complications such as anaphylaxis or secondary infection [13,14].

Distinguishing PRECs from other retroperitoneal lesions can be challenging, highlighting the importance of a comprehensive diagnostic evaluation. The differential diagnosis of PRECs includes a wide range of benign and malignant conditions, such as renal cysts, adrenal tumors, lymphomas, or retroperitoneal sarcomas. On physical examination, a palpable mass may be appreciated in the abdominal or flank region. Percussion of the abdomen may reveal dullness over the area of the cyst. However, clinical findings alone are often insufficient for an accurate diagnosis [13,15]. Imaging modalities play a crucial role in the diagnosis of PRECs. Ultrasonography, computed tomography, and magnetic resonance imaging are commonly used to visualize cystic lesions and assess their anatomical relationship with surrounding structures. Imaging features suggestive of ECs include a well-defined cystic mass with a characteristic daughter cyst or hydatid sand appearance [16,17]. In addition to imaging studies, serological tests such as enzyme-linked immunosorbent assays or indirect hemagglutination assays can aid in the diagnosis by detecting specific antibodies against Echinococcus antigens in the patient's serum [14]. Fine-needle aspiration, albeit controversial due to potential cyst rupture and allergic reactions, can provide a definitive diagnosis through fluid analysis for parasite protoscolices and scolices [18].

The management of PRECs poses several challenges due to their location and potential for complications. The goals of treatment include eradication of the parasite, prevention of recurrence, and preservation of organ function. Several management options are available, including medical therapy, percutaneous interventions, and surgical excision. Medical therapy with benzimidazole derivatives, such as albendazole or mebendazole, plays a role in the management of ECs, particularly in inoperable cases or as adjunctive therapy to surgery [17]. However, medical therapy alone is often insufficient for complete cyst resolution and is associated with variable response rates. Percutaneous treatment modalities, including puncture, aspiration, injection, reaspiration (PAIR), and percutaneous drainage, have been utilized for the management of ECs, including those located in the retroperitoneum. These minimally invasive techniques aim to decompress the cyst, instill scolicidal agents (e.g., hypertonic saline, ethanol), and induce cyst collapse. While percutaneous interventions may offer a less invasive alternative to surgery, they are associated with a risk of cyst recurrence, infection, and anaphylaxis [19]. Surgical excision remains the cornerstone of treatment for PRECs, particularly for large, symptomatic, or complicated cysts. Total cystectomy, involving complete resection of the cyst with its surrounding pericyst and any associated organ involvement, is often recommended to prevent recurrence and minimize the risk of intraoperative spillage [3].

Total cystectomy, or complete surgical excision of the echinococcal cyst, is considered the definitive treatment for PRECs, offering the best chance for cure and prevention of recurrence. This approach aims to remove the entire cyst, including the germinal layer, daughter cysts, and surrounding pericyst while minimizing the risk of intraoperative spillage and dissemination of viable parasite material [3]. The decision to perform a total cystectomy depends on several factors, including the size and location of the cyst, the involvement of adjacent structures, the presence of complications (e.g., rupture, infection), and the patient's overall health status. While total cystectomy may be technically challenging, particularly for cysts located in close proximity to vital structures such as major blood vessels or the pancreas, meticulous surgical technique and perioperative management can help minimize operative risks and optimize outcomes. Several cases have reported the effectiveness and safety of total cystectomy PRECs, with overall favorable outcomes [4-7].

Limitations and research gaps

Total cystectomy is not without limitations and potential disadvantages. Total cystectomy can be technically challenging, particularly for cysts located in close proximity to vital structures or with extensive involvement of adjacent organs, necessitating advanced surgical expertise and careful preoperative planning. The proximity of ECs to major blood vessels or delicate structures such as the pancreas increases the risk of intraoperative complications, including hemorrhage, injury to adjacent organs, or inadvertent spillage of cyst contents, which may lead to anaphylaxis or dissemination of viable parasite material [3,13]. Total cystectomy may not be feasible in all cases, particularly in resource-limited settings or in patients with significant comorbidities that preclude extensive surgical intervention, highlighting the importance of individualized treatment approaches based on patient-specific factors and disease characteristics.

While total cystectomy remains the gold standard for the management of PRECs, several unanswered questions and knowledge gaps exist, warranting further research in this field. Further studies are needed to evaluate the efficacy and safety of minimally invasive approaches, such as laparoscopic or robotic-assisted total cystectomy, compared to traditional open surgery, with a focus on perioperative outcomes, long-term efficacy, and patient satisfaction. The role of adjuvant therapies, such as intraoperative or postoperative instillation of scolicidal agents, adjunctive medical therapy with benzimidazole derivatives, or immunomodulatory agents, in preventing disease recurrence and improving long-term outcomes following total cystectomy warrants investigation in prospective clinical trials. Identification of clinical, radiological, serological, or histopathological predictors of cyst recurrence following total cystectomy may aid in risk stratification and personalized treatment planning, facilitating early detection and intervention in high-risk patients. Comparative studies evaluating the cost-effectiveness of total cystectomy versus other management options, taking into account direct healthcare costs, indirect costs related to lost productivity, and quality-adjusted life years gained, are needed to inform healthcare policy and resource allocation decisions. 

## Conclusions

PRECs represent a rare yet clinically significant manifestation of echinococcosis, posing diagnostic and management challenges due to their anatomical location and potential for complications. Total cystectomy, involving complete surgical excision of the cyst and surrounding tissue, emerges as a definitive treatment option for PRECs, offering the potential for cure and prevention of recurrence. Our systematic review highlights the limited but promising evidence supporting the efficacy and safety of total cystectomy in managing PRECs, with favorable outcomes reported in terms of low recurrence rates and minimal postoperative complications. However, challenges such as technical complexity, proximity to vital structures, and the risk of intraoperative complications underscore the importance of careful patient selection, meticulous surgical technique, and multidisciplinary management. Further research is warranted to optimize surgical approaches, explore adjuvant therapies, identify predictors of recurrence, and assess cost-effectiveness.
